# Characterizing citrullination by mass spectrometry-based proteomics

**DOI:** 10.1098/rstb.2022.0237

**Published:** 2023-11-20

**Authors:** A. S. Rebak, I. A. Hendriks, M. L. Nielsen

**Affiliations:** Proteomics Program, Novo Nordisk Foundation Center for Protein Research, Faculty of Health and Medical Sciences, University of Copenhagen, Blegdamsvej 3B, 2200 Copenhagen, Denmark

**Keywords:** citrullination, post-translational modifications, mass spectrometry, proteomics

## Abstract

Citrullination is an important post-translational modification (PTM) of arginine, known to play a role in autoimmune disorders, innate immunity response and maintenance of stem cell potency. However, citrullination remains poorly characterized and not as comprehensively understood compared to other PTMs, such as phosphorylation and ubiquitylation. High-resolution mass spectrometry (MS)-based proteomics offers a valuable approach for studying citrullination in an unbiased manner, allowing confident identification of citrullination modification sites and distinction from deamidation events on asparagine and glutamine. MS efforts have already provided valuable insights into peptidyl arginine deaminase targeting along with site-specific information of citrullination in for example synovial fluids derived from rheumatoid arthritis patients. Still, there is unrealized potential for the wider citrullination field by applying MS-based mass spectrometry approaches for proteome-wide investigations. Here we will outline contemporary methods and current challenges for studying citrullination by MS, and discuss how the development of neoteric citrullination-specific proteomics approaches still may improve our understanding of citrullination networks.

This article is part of the Theo Murphy meeting issue ‘The virtues and vices of protein citrullination’.

## 1. Introduction

Citrullination is a post-translational modification (PTM) of arginine, catalysed by a group of enzymes known as peptidyl arginine deaminases (PADIs), which convert arginine to the non-coded amino acid citrulline. Despite the conversion only resulting in a small mass shift of 0.98 Da, the modification may impart a large functional impact on the protein, as citrullination of arginine causes a loss of positive charge and change electron acceptor properties [1]. This change in physiochemical properties may alter protein structure, protein–protein interactions, and protein localization within the cell [2]. However, the implications of citrullination at the cellular level remain not fully understood. Citrullination is known to play important physiological roles in maintaining stem cell potency, during the innate immune response, and in maintaining the skin barrier [3–5]. Furthermore, dysregulation of citrullination is known to be a driving factor in rheumatoid arthritis (RA), psoriasis, and cancer [5–7].

To elucidate the functional implications of citrullination, it is crucial to be able to detect citrullination events in an unbiased manner. Furthermore, it is important to identify not only the citrullinated target proteins, but also the specific arginine residues that are targeted for citrullination. For the detection of citrullinated proteins, earlier methods included colorimetric assays relying on absorbance measurement of the chemical reaction products of the ureido group of citrulline with diacetyl monoxime [8], and later with supplementary addition of antipyrine [9]. Further adaption of this method led to the Nα-benzoyl-l-arginine ethyl easter method [10]. Although able to confirm whether proteins are citrullinated, and applicable to PADI inhibitor assays, absorbance measurements are not readily applicable to complex biological samples. This is owing to their limited overall sensitivity [11], and their inability to distinguish citrullinated proteins from contaminants such as free citrulline, urea and other ureido containing molecules, which are often present in samples and therefore may hamper readout [10].

Sensitivity of detection remains a challenge for the analysis of PTMs in complex biological samples for a number of reasons; including they often are dynamically regulated and tend to target low-abundant proteins, which collectively renders them present in samples at sub-stoichiometric levels (i.e. the percentage by which a protein is modified with a given PTM) [12]. One of the strategies to alleviate this is application of methods that specifically enrich proteins or peptides modified by PTMs, facilitating subsequent study. Such enrichment strategies are often based upon the usage of antibodies, chemical probes, or specific protein domains that specifically interact with PTMs [13]. Various analytical methods have been developed, which in combination with mass spectrometric detection are now routinely used to study a range of different PTMs, including phosphorylation [14], acetylation [15], methylation [16], glycosylation [17], ubiquitylation [18], SUMOylation [19] and ADP-ribosylation [20].

In this review we will describe contemporary strategies for studying citrullination using mass spectrometry (MS)-based proteomics, discuss advantages and limitations, and highlight potential future developments. In the context of studying citrullination at the system-wide level, development of specific enrichment strategies has remained challenging primarily owing to the limited availability of citrulline-specific antibodies and the absence of other enrichment-specific probes. While commercially available anti-citrulline antibodies have rendered it possible to study citrullination using standard molecular approaches, these antibodies are either context-dependent or display low sensitivity, which hinders the use of these for unbiased enrichment of citrullination from complex biological samples [10,11]. Enrichment by chemically modifying the citrulline and identifying the modified residue is also possible, and several methods have been used to study citrullination [21,22]. Anti-modified citrulline (AMC) antibodies specific to chemically modified citrulline are widely used for detection of citrullination in a method widely referred to as the AMC or ‘Senshu' method [23]. While successfully able to detect citrullination events, the methodology requires a highly acidic environment for the reaction to take place. This may provide challenges when employing the AMC method for studying citrullination using MS, as highly acidic conditions can result in hydrolysis of peptide backbones and alteration of existing PTMs. Currently, there are no MS-based enrichment methods routinely used for studying citrullination, rendering this PTM challenging to characterize using proteomics.

Even in highly purified samples, the ability to detect and faithfully localize a PTM on a peptide greatly depends on technological advancements in the field of MS [24–26] with modern mass spectrometric technology capable of distinguishing and sequencing peptides at a very high mass accuracy, and from nanogram (ng) amounts of input material [27]. Thus, MS has emerged as a powerful approach for characterizing PTMs as it is able to provide site-specific information of which amino acid is modified by which PTM, and without any *a priori* knowledge as to which proteins and which residues are modified. As a result, MS is the preferred approach for unbiased characterization of PTMs, especially when compared to conventional low-throughput biochemical strategies, such as immunoblotting, which only considers a limited number of proteins and is dependent on the fidelity of the antibodies used to detect them.

The information obtained from MS studies often provides valuable systemic insights, especially when studying less well-characterized PTM such as citrullination. In particular, MS-based studies can provide details into the cellular distribution of the investigated PTM, and may furthermore provide valuable biological insights into targeting preferences and biological functions of the PTM. Taken together, specific enrichment approaches combined with MS analysis represent a very powerful tool for characterizing PTMs, and we anticipate that MS-based proteomics may similarly become a method of choice for furthering our knowledge of protein citrullination.

## 2. Mass spectrometry

MS has a wide range of applications, with measurement of the mass over charge (*m/z*) ratio of any molecule as its primary ability. In the context of measuring the *m/z* of proteins and peptides at a systemic level, this is referred to as proteomics. In order to measure *m/z* values the mass spectrometer requires that the molecules of interest are first ionized; a process in which molecules are forced into a charged and gaseous state [28]. Different ionization methods have been developed to ensure efficient ionization of various molecules. In particular, the development of matrix assisted laser desorption ionization and electrospray ionization kicked off the application of MS for biomolecules such as proteins [29,30]. Different mass analyser and fragmentation technologies are also available, and detailed below, ultimately the choice of instrumentation is based on the sample, level of resolution needed and inevitably availability.

In a proteomic analysis the ionized peptides enter the MS and the *m/z* values of all peptides in the ion beam are concomitantly measured between a lower and upper *m/z* bound by the mass analyser, which represents a full scan [31]. Following this, a narrow band of the *m/z* window can then be isolated for further characterization via tandem mass spectrometry (MS/MS), which entails fragmentation of the peptide/protein to determine its amino acid sequence and facilitate localization of PTMs. Ideally peptides are fragmented across the full peptide backbone to facilitate good peptide sequencing and localization of PTMS. The peptide precursor mass is derived from the full scan by monitoring the isotopic distribution of the precursor, and along with the peptide fragment masses obtained via MS/MS, this information is subsequently compared to a database containing theoretical peptide fragmentation spectra, ultimately leading to identification of the exact peptide sequence [32].

Broadly speaking, MS-based proteomics can be divided into two main approaches, bottom-up proteomics and top-down proteomics. Bottom-up proteomics, is based on the analysis of proteins that are proteolytically digested into peptides prior to analysis, whereas top-down proteomics is the analysis of intact proteins [33,34]. Bottom-up proteomics is the most commonly used approach for PTM research, we believe it is currently the most relevant strategy for the study of citrullination and is the main strategy of the rest of this review.

## 3. Bottom-up proteomics

**Figure 1.**
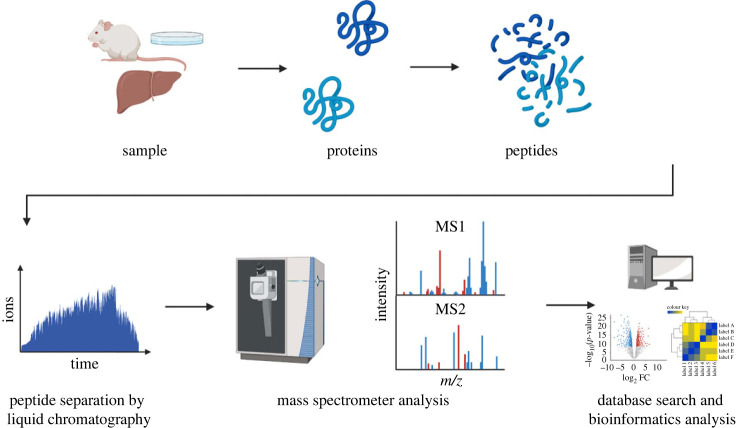
Proteins are extracted from a biological sample such as cell culture or organs and subjected to proteolytic lysis to generate peptides. The peptides are separated by reversed-phase liquid chromatography coupled to the mass spectrometer (MS) and enter the MS as ionized peptides achieved by electrospray ionization. A full scan of the peptide mixture is recorded. Precursors ions are selected for fragmentation and fragment ions are recorded to generate the MS/MS scans. The full scan and MS/MS scans are analysed to obtain peptide identification and PTM localization, and bioinformatic analysis may be performed to gain additional biological insights.

In bottom-up proteomics the material of interest such as cultured cell lines or tissue samples are harvested, lysed and homogenized before the protein mixture is proteolytically digested prior to analysis on the MS. The main advantages of bottom-up proteomics in contrast to top-down are the existence of many peptides per protein allows multiple chances at identifying proteins and peptides ionize much more readily than full proteins which improves sequence coverage, identification and quantification. Furthermore, chromatographical separation of peptides is less complicated and interpretation of peptide MS/MS spectra is simpler [28]. Figure 1 depicts a standard bottom-up proteomics workflow.

### (a) Protein digestion

For the purpose of proteolytic digestion of proteins, there is a broad spectrum of enzymes available, each with specific cleavage preferences and each with distinct niches in which they are applicable [35]. Trypsin and Lys-C are the most used in bottom-up proteomics experiments. Trypsin cleaves exclusively C-terminal to arginine and lysine residues [36], whereas Lys-C only cleaves C-terminal of lysine residues. Although Lys-C and trypsin can both cleave C-terminally of lysine residues, the combination of the two enzymes nonetheless results in a more efficient digestion compared to trypsin alone [37]. Trypsin and Lys-C are prevalently used owing to the peptide sizes generated, and because of the distribution of charged residues across the peptide, carrying a charge at the protonated N-terminus of the peptide and at the amine group of arginine or lysine residues, which enhances higher-collision energy dissociation (HCD) fragmentation and downstream peptide identification [38,39]. Trypsin cleaves at a reduced rate C-terminally of citrulline, Tran *et al*. [40] quantified the reduced cleavage using fluorogenic peptide digest. They found that the catalytic efficiency of trypsin on an arginine containing peptide was 3.9 × 10^7^ M^−1^ s^−1^ while the catalytic efficiency on a similar, but citrullinated peptide was undetectable. Some studies have used the poor cleavage efficiency following citrulline to exclude identified C-terminal citrullination sites in tryptic digests during data analysis [41,42]. Alternatively, Lys-C only digestion can be used for citrullination studies as it avoids cleavage of arginine and citrulline residues altogether [43].

### (b) Separation techniques

**Figure 2.**
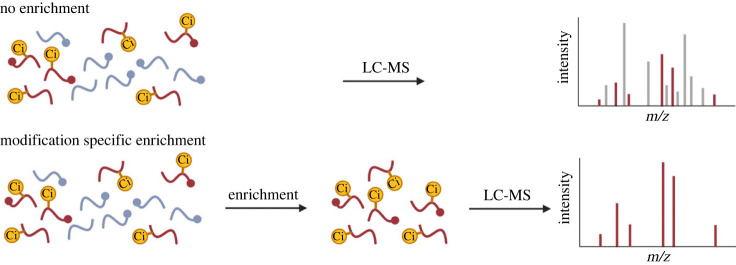
PTM enrichment allows for the analysis of only modified peptides.

Because the analytical dynamic range of a MS is finite, it is very advantageous to reduce the complexity of highly dynamic and complex biological samples, such as proteomes, in order to improve sequencing depth [44]. Online liquid chromatographic separation in combination with mass spectrometry (LC-MS) is a standard approach used to temporally reduce sample complexity and thereby provide the MS with more time to sequence peptides. In PTM research, enriching modified peptides and removing unmodified peptides prior to MS analysis results in a significant reduction of sample complexity (figure 2). Unfortunately, hitherto no well-established enrichment method exists for large-scale analysis of citrullination.

**Figure 3.**
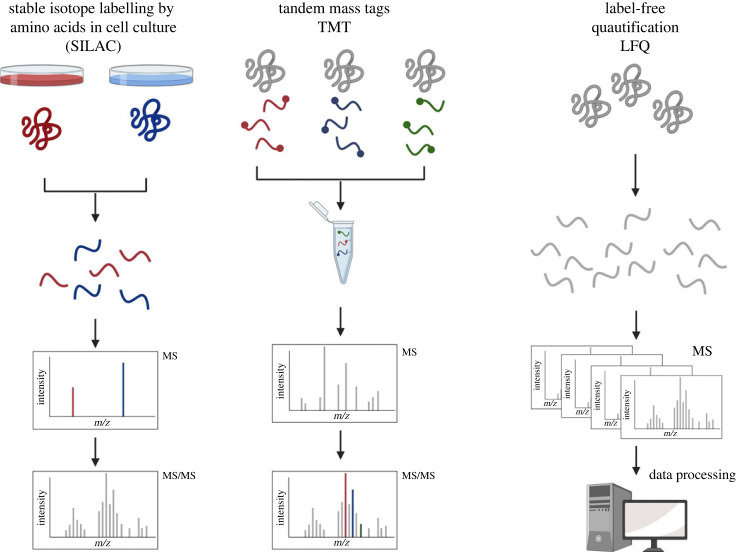
MS quantification strategies based on metabolic labelling (SILAC), chemical labelling (TMT) and label-free (LFQ). During SILAC samples are distinguished at the MS1 level according to the stable isotopic amino acid labelling. TMT labelled samples are distinguished at the MS1 level according to the stable isotopic amino acid labelling. TMT labelled samples are distinguished at the MS2 levels owing to the chemical tags resulting in different cleavage ions during fragmentation. During LFQ samples are run separately on the MS and are compared during the data processing level.

Samples may be fractionated prior to MS acquisition, also referred to as pre-fractionation or offline fractionation, to reduce sample complexity. Sub-cellular fractionation can also be used to reduce complexity by separating nuclear and cytoplasmic proteins from each other, or by isolating specific organelles, such as mitochondria prior to further sample preparation. Prior to digestion to peptides, one-dimensional gel-separation by electrophoresis can separate proteins based on the charge and mass [45]. In the field of PTM research, peptides are often pre-fractionated, commonly by reversed-phase liquid chromatography. For example, C_18_ StageTips can be used to fractionate samples [46], or alternatively high performance liquid chromatography (HPLC) can be used to the same effect [47], separating peptides based on their hydrophobicity at high pH. Strong cation exchange is another prevalent separation technique separating peptides based on charge and mass [48,49]. Typically the peptides are further separated by online reversed-phase HPLC fractionation, which separates the peptides based on their hydrophobicity at low pH [50]. The HPLC column contains a stationary phase made of C18 material that binds peptides based on their hydrophobicity. An increasing concentration of organic solvent, such as acetonitrile, causes peptides to elute in a gradient from most hydrophilic to most hydrophobic [51]. Importantly, if using several separation techniques, it is most advantageous if they separate peptides based on independent and orthogonal properties in order to increase the separation achieved and as a result improve the identification [52].

### (c) Fragmentation techniques

Once a full scan has been performed across the entire *m/z* range, precursor ions are selected and isolated via the quadrupole, after which ions are sent for fragmentation. The aim of fragmentation is to break the peptide backbone between amino acids, with the breaks distributed across the entire backbone, making it possible to sequence the full peptide and localize PTMs. Fragmentation is also crucial for the generation of charged losses (also referred to as diagnostic peaks) or neutral losses that can be used to fingerprint and validate specific PTMs, such as citrullination [53], SUMOylation [19] and phosphorylation [54,55]. Precursor ions may be selected on a TopN method basis, where the *n* number of most abundant ions are selected for fragmentation, which is also known as data-dependent acquisition, as it relies on live data derived from full scans. Alternatively, data-independent acquisition (DIA) MS/MS scans are performed across predefined, small and overlapping *m/z* windows which isolate ions indiscriminately across the full range [56].

Collision-induced dissociation (CID) is a traditional method of ion dissociation. Here the precursor ion collide with an inert gas, such as nitrogen, helium or argon, which causes fragmentation of the peptide backbone generating *b-* and *y-*type fragment ions [57,58]. Limitations of CID include poor characterization of labile PTMs as these are lost during fragmentation, and poor characterization of ions of low *m/z* [59]. The development of HCD alleviated most of these shortcomings by using a higher energy fragmentation and generating more informative fragment ions such as diagnostic ions used for PTM identification. HCD can localize labile PTMs better than CID, and ions across the full-mass-range can be fragmented through the introduction of a c-trap [60].

A separate technology, electron-transfer dissociation (ETD), relies on peptides gaining excess energy from electrons removing one positive charge from the peptides. The fragmentation often occurs at the peptide backbone without loss of any labile PTMs. This type of fragmentation generates *c-* and *z-*type ions [61,62].

Electron-transfer and higher-collision dissociation (EThcD) combines ETD-type dissociation with HCD. Here ions are first subjected to ETD, after which the ion package is transferred to an HCD cell and subjected to HCD fragmentation. The spectrum produced is a mix of *b/y-* and *c/z-*ions. The total time it takes to make a scan is increased somewhat, however, peptide sequence coverage and identification can be substantially improved [63], especially when analysing large peptides or proteins, or in cases where the ETD reaction does not lead to spontaneous fragmentation of the peptide. A combination of fragmentation techniques can also use for targeted approaches where signature ion masses are detected using rapid HCD or CID scans, which then triggers a more sensitive ETD fragmentation of the precursor of interest. Such methods have been applied in the context of MS-based study of citrullination [64,65].

## 4. Quantitative proteomics: stable isotope labelling by amino acids in cell culture, tandem mass tags, label-free

MS not only allows for confident identification of peptides and proteins, but also enables the absolute or relative quantification of protein levels across different samples. This is very valuable when evaluating temporal changes in response to cellular stresses or treatments [31,66,67]. In terms of the quantification of PTMs, researchers are often interested in the extent of site modification, the fraction of proteins containing the modification at a specific site, referred to as PTM site stoichiometry or occupancy, instead of site intensity only. The site stoichiometry in combination with the temporal changes in response to treatment may reveal biological significance. For example, a 10-fold increase in site intensity can be a result of 2–20% site stoichiometry or 10–100%, which maybe have different cellular effects [12]. It should further be noted that the detected site occupancy may also be affected by the overall level of protein for example through partial protein degradation, in addition to peptide detection rates.

A range of different approaches exist for relative quantification of peptide and protein changes, including metabolic isotopic labelling such a stable isotope labelling by amino acids in cell culture (SILAC), chemical isotopic labelling such a isotope-coded affinity tags [68] and isobaric tagging such as isobaric tag for relative and absolute quantification [69] and tandem mass tags (TMT) [70]. Figure 3 depicts MS quantification strategies based on metabolic labelling (SILAC), chemical labelling (TMT) and label-free quantification (LFQ).

SILAC is applicable to the analysis of cell cultures which grow normally using dialysed serum. Stable isotope-labelled arginines or lysines are added to the cell-culture medium which lacks naturally occurring arginines or lysines, and the labelled amino acids are then incorporated into the proteome of the cell population. Cells can either be light labelled (also referred to as unlabelled), medium labelled or heavy labelled. Cell lysates from two or three differentially labelled, and usually differentially treated, cell populations are then combined and relative protein levels can be quantified based on the ratios of the differentially labelled peptides [71,72]. This approach allows samples to be mixed at a very early stage, and thus they are treated equally and technical variance is minimized. Furthermore, since differentially labelled peptides do not exhibit different chromatographic characteristics, the labelled duplets or triplets will always co-elute allowing for reliable and precise quantification.

Peptides can also be labelled at a later stage during sample preparation, following digestion of proteins and purification of the resulting peptides. This is for example relevant for studying human biopsies, which cannot be metabolically labelled, or in case of cell cultures that cannot grow with dialysed serum in the growth medium. One example of peptide mass tag labelling is TMT, where samples are chemically labelled *in vitro*, allowing any protein to be labelled [70,73]. Peptide fragmentation dissociates the TMT tag giving rise to a fragment ion of a particular *m/z*. Although the number of metabolic or chemical labelling channels is limited, it is possible to design an experiment containing a greater number of experimental conditions compared to the number of labels, by generating several multiplexes that all include one common control channel, which can then be used to normalize the different multiplexes to each other [74].

Label-free approaches are becoming increasingly common, owing to the simpler design and sample-preparation methods. New developments in computational methods have greatly improved the precision in determining peptide ratios based directly on peptide signal [75].

Absolute quantification is also possible and traditionally relies on the use of labelled peptides that are spiked into samples, where they act as internal standards with known concentrations, and by comparison enable determination of absolute protein concentrations [69,76].

## 5. Bioinformatics analysis of proteomics

MS data require robust computational tools to process. Typically, it involves interpretation of the raw data generated by the instrument, tracing precursors over *m/z* and time dimensions, accurate determination of charge and mass, filtering and interpretation of the MS/MS spectra, comparison of peptide mass and fragmentation patterns to theoretical entries in an *in silico* digest of the relevant species, mapping any identified peptides to corresponding proteins and finally quantification of the proteins.

Comparison of acquired MS/MS spectra to a library of theoretical spectra derived via *in silico* digestion of proteins from the organism of interest yields a score which is based on how many peaks match between practical and theoretical spectra. The highest scoring match, provided it is above a certain threshold, is known as a peptide spectrum match (PSM) [32]. There are many data processing tools capable of doing this, including the commonly used software MaxQuant which makes use of the Andromeda search engine [77,78]. Other common data search engines include Mascot [79] and SEQUEST [80]. Most search engines use a target-decoy-strategy to allow for false discovery rate (FDR) calculations, and subsequent filtering of PSMs to limit the number of false positive hits below 1% [81] at the PSM level. MaxQuant additionally applies FDR control at the protein level, as well as at the site decoy level in case of PTMs. Modifications such a citrullination are specified as variable modifications prior to the data processing, which allows the search engine to consider both unmodified and modified versions of peptides. The specification of modifications increases the size of the *in silico* database and herewith increases the search time [78]. In the case of citrullination, the variable modification used for proteomic data searches essentially corresponds to a deamidation of the arginine. For this, it is important to simultaneously also include deamidation of asparagine and glutamine in the database search approaches as these modifications commonly are introduced during MS sample preparation. Deamidation of all three residue types must therefore be considered to avoid potential artificial localization of the mass shift to the wrong amino acid residue by automated algorithms, although sufficiently high-quality MS/MS spectra along with careful evaluation of acquired spectra prevent ambiguous localization [82].

The output of search engines is usually an overview of all PSMs, identified peptide sequences, and the proteins these can be mapped to, along with qualitative and quantitative information. These data can be interpreted using a range of software. Perseus is a bioinformatics tool used by many proteomics researchers to further analyse the search engine output data, and can perform a wide range of statistical tests and visualizations to assist in interpreting, e.g. proteome changes [83]. Many other specialized bioinformatics tools exist, such as the web-based STRING tool, which is capable of interpreting lists of proteins or gene names, and generates protein interaction networks based on existing knowledge mined from the scientific literature [84].

## 6. Studying post-translational modifications systems-wide: advantages, challenges and current state of the art

Studying PTMs systems-wide provides invaluable insight into overall cell signalling, and facilitates the understanding of PTM function in this context [13]. By identifying which PTMs affect which residues in which proteins, researchers can shed light on e.g. how these PTMs affect protein–protein interactions [85], or how they affect chromatin structure [86] and downstream gene expression *in vivo* [3], protein turnover [87], protein localization [88] and regulate enzymatic kinetics [89]. While MS screens are great as exploratory step to direct follow-up research, it is also a powerful technology that may give biological insight without *a priori* knowledge of the system. Recent MS efforts for example uncovered the organization of the histone chaperone network important for regulation of gene silencing [90], while MS studies of SUMOylation have shown that SUMOylation plays a significant role in repairing DNA–protein cross-links [91] and identified crosstalk between SUMOylation and phosphorylation [92].

The main challenge of studying PTMs with MS is the low abundance of PTM-modified peptides relative to other peptides, which makes it more challenging to identify PTMs because MS precursor selection is inherently a stochastic process driven primarily by abundance [13,93]. For many PTMs, enrichment methods have alleviated this challenge. Large amounts of starting material are often necessary to allow a sufficient amount of PTMs, such as ADP-ribosylation [94] and SUMOylation [92] to be enriched, as these modified peptides are often many of orders of magnitude less abundant compared to unmodified peptides. Multiple modifications exist with very similar mass shift, such as the case for citrullination and deamidation of asparagine and glutamine and correction identification of these require very high resolution to distinguish the mDa differences. Additionally, some PTMs are biologically, chemically or physically labile, and can be lost during cell treatment, sample preparation or peptide fragmentation during MS/MS, respectively. For faithful identification and localization of PTMs on a peptide, a high degree of sequence coverage is required, with either a part of or the entire PTM still residing on the corresponding amino acid [13].

## 7. Mass spectrometry for citrullination and the challenges associated

**Figure 4.**
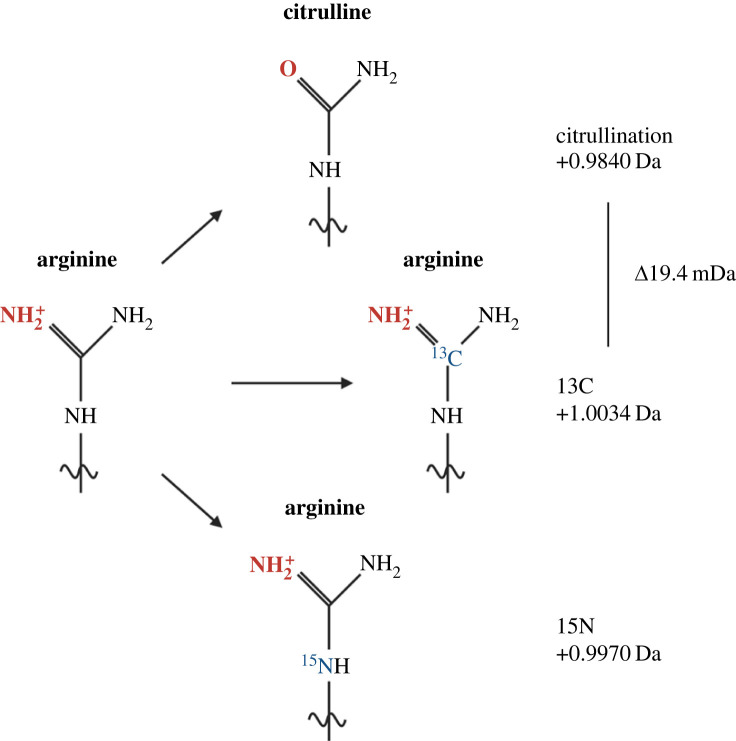
The mass shift caused by citrullination is very similar to the naturally occurring shift caused of carbon-13 (^13^C) and nitrogen-15 substitution of the arginine residue.

While MS is an excellent technology for studying citrullination, there are associated challenges that should be considered when designing a proteomics experiment. It is important to employ a high-resolution mass analyser, for example an Orbitrap instrument, in order to achieve a mass accuracy that is sufficiently high to distinguish citrullination (+0.9840) from the naturally occurring isotopes ^13^C and ^15^N on arginine causing a mass shift of +1.0034 Da and +0.9970 Da, respectively. The mass difference between a citrullinated arginine and an arginine with stable isotope ^13^C is just 19.4 mDa (figure 4). Hence, low mass accuracy could result in incorrect assignment of ^13^C containing arginine residues as citrullinated residues, and thus represent false positive identification [95,96].

## 8. Contemporary mass spectrometry strategies for studying citrullination

### (a) Diagnostic markers for validation of citrullination

In addition to identifying the expected citrulline mass shift to a specific arginine residue, additional information can be derived from the fragmentation of citrullinated peptides.

**Figure 5.**
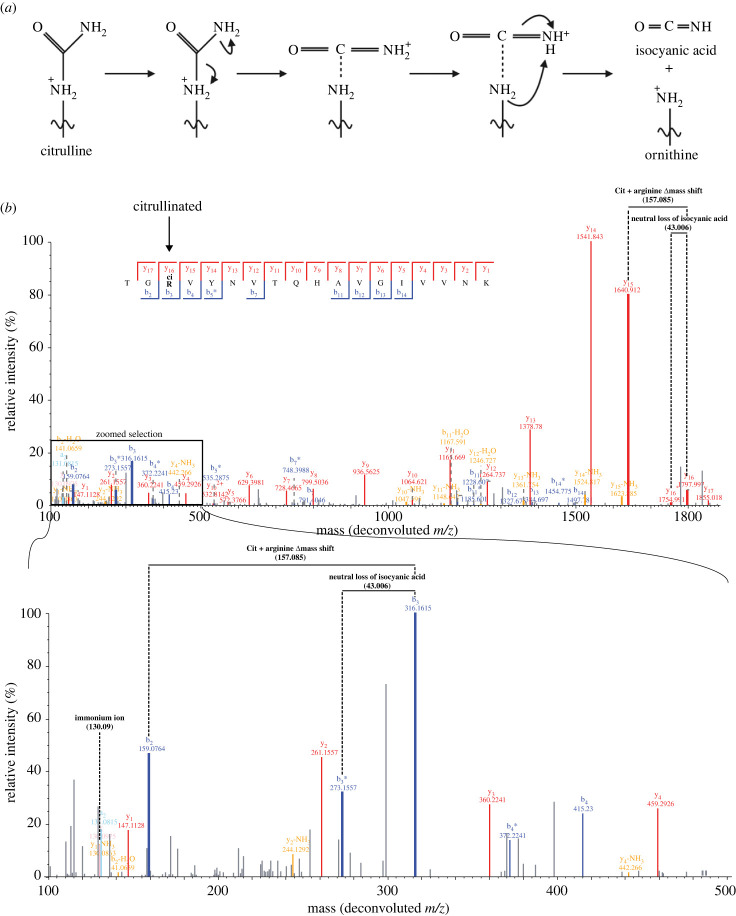
(*a*) Loss of isocyanic acid from citrulline peptide during fragmentation causing neutral loss of 43.006 Da. (*b*) Annotated MS/MS spectrum of citrulline-containing peptide and zoomed selection, demonstrating the mass shift of citrulline from b_2_ to b_3_ ion and matching y_15_ and y_16_, neutral loss of isocyanic acid from the citrulline b_3_ to b_3*_ and similarly from y_16_ to y_16*_. Additionally, the immonium ion of citrulline at 130.09 *m/z* is also detected and highlighted. Blue, b-ions; red, y-ions; orange, z-ions; grey, unassigned. Spectra obtained from Rebak *et al*. [43].

By studying synthetic citrulline-containing peptides Hao and colleagues found an abundant neutral loss of 43 Da, in CID spectra from citrullinated peptide precursor ions. They matched this neutral loss to the loss of isocyanic acid, HNCO, from the citrulline ureido group, occurring in multiple charge states of the precursor ion, in b*-* and y-ions [53] figure 5. Including the neutral loss in the search of MS/MS data increases the confidence when matching spectra and reduces the number of false positives, thereby improving the discovery of novel citrullination sites [53]. The neutral loss of isocyanic acid is now commonly used during data searches to improve citrullination identification rates [65,97,98]. Recently, a method was published that optimized the abundance of the isocyanic acid loss, by using stepwise collision energy to achieve a higher peptide backbone coverage [99]. A diagnostic ion used to validate the presence of a citrullination event is the immonium ion of citrulline, which can be observed at 130.0975 Da in MS/MS spectra. Immonium ions are a product of multiple backbone fragmentations of a peptide, resulting in a fragment containing just one amino acid, which results in an ion that is 27 Da smaller than the amino acid [100]. Immonium ions are also used as diagnostic ions to improve the detection confidence of for example phosphotyrosine [101].

MS data search algorithms can be designed to account for the neutral loss of isocyanic acid and occurrence of the citrulline immonium ion. The ionFinder algorithm as developed by Maurais *et al*. [102] developed two algorithms for rapidly identifying citrullinated peptides abased on the presence of the neutral loss of isocyanic acid. The group also made the EnvoMatch algorithm available which improves identification of citrullinated peptides based on the small mass shift of citrulline.

When mining published proteome spectra, Lee *et al*. found that neutral loss of isocyanic acid is a reliable way to distinguish citrullination of arginine from deamidated Asn and Gln residues. However, they also found that the immonium ion of citrulline is not a good diagnostic ion, but that spectra containing the citrulline immonium ion have a higher validation rate compared to spectra that do not. Lee *et al*. [82] identified 375 citrullination sites on 209 proteins and found that citrullination levels do not correlate with PADI expression levels, indicating differential regulation in different tissues. This may also be owing to the immonium ion overall constituting a lower fraction of total MS/MS intensities and therefore may only be visible in more abundant citrullination events. Notably, the investigated sites were predominantly found on abundant proteins, underlining a limited depth of sequencing, and emphasizing the need for a citrullination-specific methodologies for proteome-wide investigations into citrullination and the occurrence of immonium ions.

**Figure 6.**
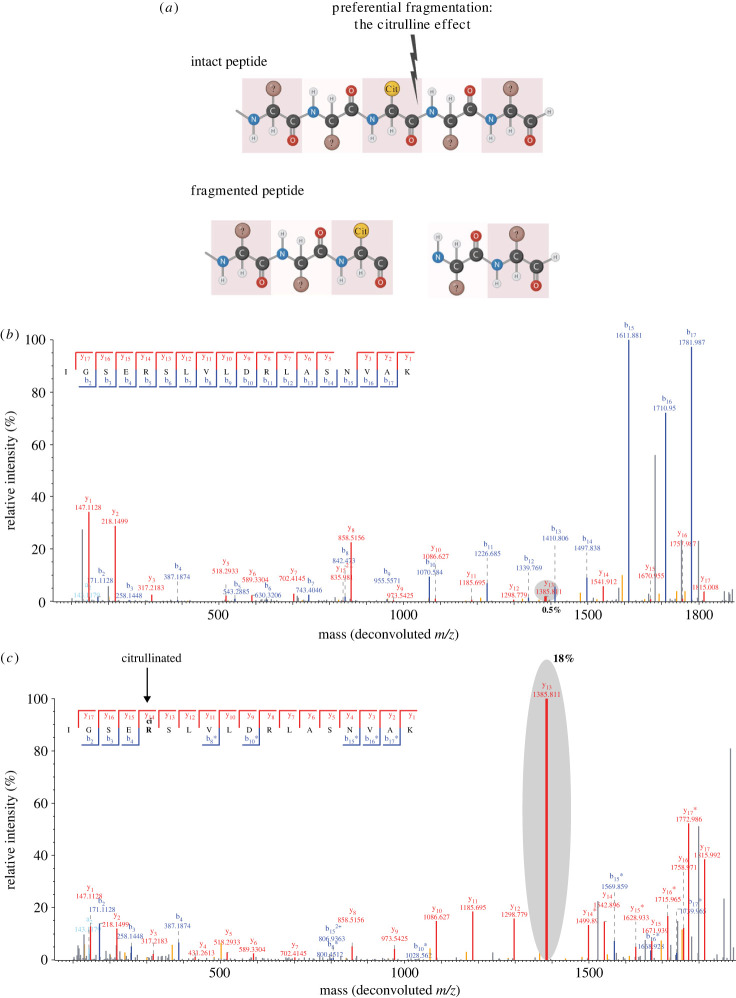
(*a*) Illustration of the citrulline effect which visualizes increased propensity of fragmentation following citrulline. (*b*) Baseline unmodified peptide. The y_13_ ion contributes 0.5% to the total ion intensity. (*c*) Citrullinated peptide demonstrating the citrulline effect, exemplified by y_13_ ion contributing 18% of the total ion intensity.

Steckel *et al*. [103] also found that the neutral loss of isocyanic acid is a frequent during fragmentation of citrullinated peptides exposed to low-energy CID. They further identified an alternate fragmentation event they coined the citrulline effect, which is the preferential fragmentation N-terminal of a citrulline positioned at the peptide C-terminus, thereby producing characteristic y_1_ ions (figure 6*a*). This is similar to the proline effect; the preferential cleavage N-terminal of proline residues [104], which is slightly stronger than the citrulline effect [105]. Nonetheless, Steckel *et al*. recommend that the citrulline effect is used as a complementary validation of deiminated peptides, and showed that the citrulline effect occurred in 44% of the citrulline-containing peptides found in the proteome mining study by Lee *et al*. [82,105].

Choi *et al*. used the reaction between citrulline and 4-bromophenyl glyoxal [4BPG] to label citrullinated peptides. The bromine signature was detected by MADLI-TOF MS and used to localize four and five citrullination sites on bovine serum albumin and bovine fibrinogen, respectively [106].

### (b) Data search-assisted approaches

Alternative data search approaches have been proposed to improve identification and confidence of the citrullination sites detected. A dual-search Delta Score strategy was proposed by Wang *et al*. to improve the confidence in identification. This strategy relies on two separate data searches, one with and one without the variable modification of +0.9840 Da on asparagine, glutamine and arginine (NQR), and then looking for score deltas greater than zero when comparing the same spectrum across both searches, resulting in a final citrullinated PSM FDR of approximately 2% [96]. It should be noted, that use of the MaxQuant data suite including the Andromeda search engine inherently performs similar operations for all modified peptide variants detected [78].

The low rate of tryptic cleavage C-terminal of citrullinated arginine residues has been used in several studies as justification for excluding any peptides identified to contain c-terminal citrulline [41,98]. It is not clear whether this inefficient tryptic cleavage is owing to citrulline lacking the positive charge normally present on arginine [107]. However, the fact that some tryptic activity may be possible towards citrullinated arginines could still be considered in data searches, although extensive validation of C-terminal citrullination events should be performed to rule out mislocalization.

### (c) Enrichment approaches

Enrichment methods greatly reduce sample complexity while concomitantly increasing the prevalence of PTM-modified peptides. PTM-modified peptides are typically enriched using antibodies that recognize the PTM, such lysine acetylation [108] and arginine methylation [109], protein domains that have high affinity for the PTM such as ADP-ribosylation [20], enrichment using metal-affinity, or by chemically modifying a PTM and enriching for this. Anti-citrulline antibodies are commercially available, and have been used to enriched citrullinated peptides from synovial fluid of RA patients to identity 200 sites [110]. However, most of the available antibodies display a bias towards amino acid context in addition to the presence of a citrulline, which can skew the profiling of citrullination sites. Additionally, poor selectivity makes antibodies inefficient for enriching citrullinated peptides from complex biological samples such as full cell lysates or plasma [10,11]. For antibodies to be routinely used for enrichment of citrullination, new antibodies need to be developed.

The ureido group of citrulline can chemically modified to facilitate enrichment of citrullinated peptides. Glyoxal derivatives react with citrulline under acidic conditions, and have been used to detect citrulline. Adding a chemical moiety to citrulline creates a larger mass shift than the 1 Da shift caused by citrullination equal to the shift caused by deamidation asparagine. A phenylglyoxal-based probe was used for the detection of citrulline with a very low limit of detection (approx. 20 fmol citrullinated PADI4), and is suitable for high throughput colorimetric visualization of protein citrullination, however without specific site identification [111]. Lewallen *et al*. [112] developed a biotin-conjugated phenylglyoxal probe (biotin-PG), which they used to enrich citrullinated proteins from HEK293T cell overexpressing PADI2, and identified 50 citrullinated proteins by mass spectrometry. While the probe successfully detected citrullinated proteins from complex samples, it does not identify which arginine residues are citrullinated [112]. The biotin-PG was combined with on-bead tryptic digestion of citrullinated proteins enriched from RA patient serum and synovial fluids, and used to characterize the RA-associated citrullinome. These data were used to guide further research into the effect of citrullination on serine protease inhibitors (Serpins), with citrullination found to abolish the inhibitory activity of Serpins and thereby activating targeted proteases [113].

Tutturen *et al*. [114] developed a bead-based phenylglyoxal-rhodamine probe to detect citrulline-containing peptides from the myelin basic protein. However, the method was not sensitive enough for complex samples where numerous competing peptides are present. Further development by Tutturen *et al*. [21] led to an enrichment method dependent on biotin-labelled 4-glyoxalbezoic acid (BPG), which reacts with the ureido group of citrulline but is only specific under strong acidic conditions. Citrullinated peptides were enriched using streptavidin pulldown, and detected via a signature *m/z* 270 ion resulting from the HCD fragmentation of BPG [21]. This facilitated detection of 150 unique citrullination sites from synovial fluid. However, the authors reported that HCD fragmentation induces fragmentation of BPG, with most of the fragmentation energy directed to generate BPG fragment ions rather than peptide backbone ions. This resulted in MS/MS spectra with limited peptide fragmentation and in turn reduced peptide fragmentation coverage. A modification of BPG by the addition of a pH-dependent cleavable site was suggested to remove BPG prior to MS analysis and circumvent the fragmentation issues [21].

Citrullinated peptides can be chemically labelled using either 2,3-butanedione alone or in combination with antipyrine, resulting in a 50 Da or 238 Da shift, respectively [9,22]. The reaction with 2,3-butanedione and antipyrine is used in the Senshu method, where citrulline is modified and subsequently detected using an AMC antibody [23,115]. When applying the Senshu method to a mixture of synthetic citrullinated fibrinogen peptides the reaction was not complete, but allowed for the detection of 15 citrullination sites [22].

### (d) Total proteome approaches

Total proteome approaches can be used for characterization of PTMs and offer an alternative to enrichment approaches. The application of high-resolution MS in combination with pre-fractionation to increase the depth of analysis allows characterization of citrullination in a system-wide manner from total proteome samples. Raijmakers *et al*. [76] analysed whole synovial fluid from RA patients, and found high levels of fibrinogen-derived citrullinated peptides compared to healthy controls.

Because citrullination naturally occurs at low levels, characterization of citrullination from total lysates can be facilitated by addition of a recombinant PADI enzyme, which drives the citrullination of target peptides *in vitro* [116,117]. SWATH-MS, a DIA method based on a spectral library of citrullination-specific peptides, was used to identify 304 citrullination sites on 145 cardiac proteins incubated with PADI2 [117]. In a more recent study, mouse tissue homogenates were incubated with a recombinant PADI enzyme, which facilitated *in vitro* citrullination and led to a large expansion in the number of published citrullination sites [116]. While this approach is applicable to disentangle PADI activity and specificity, the resulting citrullination sites were induced *in vitro* and may therefore not fully constitute physiologically events.

To map physiological citrullination sites, we mapped citrullination in the HL60 cell line, where PADI4-specific citrullination was induced by addition of calcium ionophore and optionally inhibited by addition of the PADI4-specific inhibitor GSK484. Offline high pH fractionation was used to split Lys-C digests of total cell lysates into 46 fractions, and followed by high-resolution LC-MS/MS analysis. This work facilitated systems-wide characterization of more than 14 000 citrullination sites, hugely expanding the library of known citrullination sites, demonstrating widespread modification including extensive modification of transcription factors and chromatin remodelling factors, as well as differential regulation of citrullination at known histone marks in response to the inhibitor. Moreover, the citrulline effect was routinely observed in the data from Rebak *et al*., as exemplified in figure 6*b,c* [43].

### (e) Targeted fragmentation approaches

Targeted fragmentation approaches refer to MS methods where observation of fragment ions with specific properties, such as a specific mass or charge, trigger fragmentation of the precursor by an alternative fragmentation technique. The combination of different fragmentation techniques, such as CID triggering ETD, can improve peptide coverage and are crucial in characterizing fragment ions that may not fragment well using either one or the other fragmentation technique [118]. Targeted proteomics is used in detection ADP-ribosylation where EThcD is commonly used [119], with time this may also be the case in the field of citrullination.

In the field of citrullination, targeted approaches have been described, and can be used in combination with enrichment approaches or in total proteome approaches [64,97]. Stensland *et al*. used a targeted method for detecting citrulline modified by 2,3-butanedione and antipyrine, and showed that CID fragmentation consistently produced a highly abundant ion at *m/z* 201.1, which matches the ions mass of the modification fragment. However, CID does not always induce complete peptide backbone fragmentation, which can result in lower sequence coverage. However, they found that ETD fragmentation leaves an intact chemical modification on the citrullinated residue, and generates high-quality spectra. Stensland *et al*. performed alternating CID and ETD scans of precursors, and demonstrated increased detection of citrullinated peptides compared to using CID only [64].

The neutral loss of isocyanic acid from citrullination peptides during CID has been used to trigger subsequent ETD scans of the same precursor, and this targeted approach was found to improve identification compared to CID alone [65]. Jin *et al*. [97] developed a method based on the neutral loss from CID triggering HCD fragmentation to identify three novel substrates for citrullination in brain samples.

While targeted approaches improve identification of citrullination, it should be noted that introduction of additional fragmentation steps impairs duty cycle, affects dynamic range and in turn overall analytical sensitivity.

## 9. Conclusion and future perspectives

While there exist robust MS-based proteomic workflows for the rigorous identification and quantification of various PTMs, methodologies for comprehensive characterization of citrullination remains challenging. Although MS is an appropriate technology for studying PTMs at a systems-level, several analytical challenges associated with studying citrullination has hitherto limited systems-wide insights into this important protein modification. To achieve this, large-scale citrullination studies still need to be made faster, more sensitive and more reproducible. Hence, we foresee that in the near future, many citrullination projects will start with a high-resolution and quantitative proteomics screen of citrullination events, which will provide the basis for functional hypotheses. These hypotheses are then followed up either with standard functional assays or in combination with quantitative proteomics methods. For this, quantification will become a valuable tool in order to reduce the number of citrullination events selected for experimental investigation to a manageable number. Moreover, with increasing availability of the technology to signalling biologists, the large-scale PTM quantification resources generated by todays MS analyses will increase the likelihood that the acquired data will be functionally validated by detailed study of key modification sites. As a result, we expect that in the next few years, instrumental developments, improved protocols and computational tools will all work together to make this vision a reality. Similarly, recent developments in multiplexing strategies [66,120] will probably become important for upcoming citrullination studies in order to address the challenges of analysing larger number of samples, for example, from patient cohorts or animal material. With such advances, large-scale citrullination analysis may soon be ready to make a direct impact in the clinic.

## Glossary

Neutral loss: During fragmentation the typically doubly or triply charged precursor ions are dissociated into fragments each carrying at least one charge. Fragmentation may alternatively result in a charged fragment and a neutral fragment, this is known as a neutral loss. Owing to the neutral charge of the neutral loss, it is not visible, but the loss of mass is visible on the remaining peptide backbone.
Immonium ion: An immonium ion is the product of an internal fragment ion containing a single amino acid side chain. The mass of an immonium ion is 27 Da less than the residue mass.

## Data Availability

This article has no additional data.
